# Umbilical cord blood mononuclear cell therapy induces clinical remission of steroid-dependent or -resistant ulcerative colitis patients

**DOI:** 10.18632/oncotarget.24541

**Published:** 2018-01-04

**Authors:** Liang Chen, Yuan Gao, Zongmei Zhang, Mingming Sun, Wenjing Yang, Zhanju Liu, Xueliang Jiang

**Affiliations:** ^1^ Department of Gastroenterology, The Shanghai Tenth People’s Hospital, Tongji University, Shanghai, China; ^2^ Department of Digestive Center, The Second Affiliated Hospital of Shandong University of Traditional Chinese Medicine, Jinan, China; ^3^ Department of Gastroenterology, Chinese PLA General Hospital of Jinan Military Command, Jinan, China

**Keywords:** umbilical cord blood mononuclear cells, azathioprine, steroid-dependent or -resistant ulcerative colitis, clinical remission

## Abstract

To compare the efficacy and safety of umbilical cord blood mononuclear cells (CBMNC) and azathioprine (AZA) in the treatment of patients with steroid-dependent or -resistant ulcerative colitis. One hundred and six patients diagnosed with steroid-dependent or -resistant ulcerative colitis were studied retrospectively, including 36 patients treated with CBMNC and 70 treated with AZA. To reduce confounding bias due to retrospective nature of this study, the propensity score matching system was applied to equipoise the pretreatment data of two groups. After matching, 35 matched pairs (1:1) were created. The ratios of clinical remission, clinical response and endoscopic mucosal healing, Mayo score, and major complications were compared between two groups at weeks 8, 16, and 36 after treatment. The results demonstrated that the ratios of clinical remission (80% *vs.* 57%, *P* < 0.05) and mucosal healing (74% *vs.* 51%, *P* < 0.05) were significantly higher in CBMNC-treated patients compared with those in AZA-treated patients at week 8. The erythrocyte sedimentation rate was significantly decreased in CBMNC group compared with that in AZA-treated group (14.5 ± 3.9 mm/h *vs.* 18.0 ± 5.7 mm/h, *P* < 0.01) at week 8. In AZA group, 2 patients had neutropenia and 3 patients had elevated alanine aminotransferase levels, whereas no obvious side-effects were observed in CBMNC-treated group. Our results reveal that CBMNC therapy appears to be an effective and safe strategy for patients with steroid-dependent or -resistant ulcerative colitis. Further prospective studies are needed to define the potential roles and mechanisms of CBMNC in the treatment of refractory ulcerative colitis.

## INTRODUCTION

Ulcerative colitis (UC) is a common gastroenterological disorder which is characterized by chronic progressive inflammatory diseases in the gastrointestinal tract. Although the exact causes of UC remain unclear, recent studies showed that UC appears to be a disorder of the host immune response to microbiota which manifested by a state of local immune hyperactivity [1, 2]. It is also widely accepted that genetic and environmental factors are involved in the pathogenesis of UC [3, 4]. Finally, all these factors lead to the infiltration of leukocytes and lymphocytes into the intestinal mucosa which is believed to be critical in the formation of mucosal lesions in UC patients [5].

During the last decade, studies from countries of Europe, North America and Asia have shown that the annual incidence of UC is 24.3/100,000, 19.2/100,000, and 6.3/100,000, respectively [6]. To date, the established therapies for UC include 5-aminosalicylates, corticosteroids and immunosuppressants. The adverse effect is major limitation in the use of corticosteroids, especially for the steroid-dependent or -resistant UC patients. Although immunosuppressants such as azathioprine (AZA) or 6-mercaptopurine are dominant drugs most frequently used in inducing and maintaining remission in refractory UC patients, there is still about 30% of UC patients dropped the AZA therapy due to the side-effects or lack of clinical efficacy [7, 8]. Recently, biological agents such as infliximab and adalimumab provide a new therapy for inflammatory bowel disease (IBD), but the looming risks including opportunistic infection and malignancy, specifically, lymphoma limit their clinical application [9]. Some refractory UC like steroid-dependent or -resistant UC patients even face a significant long-term risk of colectomy [10]. Thus, new optimal therapies aim at a cure for UC are warranted, which should not only focus on blocking intestinal mucosal inflammation, but also enhancing intestinal mucosal proliferation and coordinately remodeling during the ulceration-healing process [11].

Stem cells (SC) are emerging as a promisingly therapeutic approach for the treatment of active UC owing to its self-renewal, multipotency, immunosuppressive and tissue-repair promotion functions [12, 13]. Umbilical cord blood mononuclear cells (CBMNC) comprising a mix of monocytes, immature lymphocytes, endothelial, hematopoietic and mesenchymal stem cells (MSC) [14, 15] have been successfully applied in hematopoietic malignancy for almost 2 decades [16–18]. Their advantages including economic and simple retrieval, enrichment of hematopoietic progenitors, enhanced proliferation rate [19], and weak cellular immune responses *in vivo* [20, 21] make them a promising cellular treatment for UC. To date, emerging evidence suggests that adult SC contribute to tissue regeneration by enhancing microcirculation in murine IBD model [22]. Currently, clinical trials mainly focus on SC treatment for refractory CD and perianal fistulizing CD, only a few clinical trials focus on UC were reported [23, 24]. Based on the above evidences, we hypothesized that the CBMNC therapy to be an effective and safe strategy for patients with refractory UC. Therefore, we conducted this study to investigate the efficacy and safety of CBMNC therapy for the treatment of steroid-dependent or -resistant UC patients.

## RESULTS

### Baseline characteristics

106 patients who were diagnosed with steroid-dependent or -resistant UC in Chinese PLA General Hospital of Jinan Military Command were randomly spilt into CBMNC-treated group and AZA-treated group according to their initial treatment strategy. To reduce sample selection bias, propensity score matching (PSM) was applied to equipoise the pretreatment data of these two groups. After performing PSM for the entire population, 35 matched pairs were created. The CBMNC group consisted of 19 female (mean age, 35.4 years; age range 20–50 years) and 16 male (mean age, 37.6 years; age range 18–64 years). The AZA-treated group consisted of 20 female (mean age, 35.8 years; age range 18–65 years) and 15 male (mean age, 38.3 years; age range 20–65 years). At the initial enrollment, baseline demographics and disease characteristic were similar in both propensity-matched groups (Table 1).

**Table 1:** Baseline demographics and disease characteristics for all patients in CBMNC Group and AZA Group

Characteristics	Before matching			After matching		
	CBMNC	AZA	*P* value	CBMNC	AZA	*P* value
Total	35	59		35	35	
Male/Female	16/19	36/23	0.15	16/19	15/20	0.34
Age (years)	36.4 ± 11.1	33.7 ± 10.6	0.43	36.4 ± 11.1	30.2 ± 8.2	0.11
Course (years)	3.2 ± 1.6	3.3 ± 1.6	0.07	3.2 ± 1.6	3.3 ± 1.5	0.10
Disease extent			0.29			0.89
Proctitis	2	5		2	2	
Left-sided colitis	17	19		17	15	
Pancolitis	16	35		16	18	
Prior treatment			0.51			0.95
Corticosteroids	30	59		30	30	
Corticosteroids (≥ 20 mg/d)	17	22		17	15	
Mesalazine	30	59		30	30	
Intestinal surgery	0	0		0	0	
Baseline rectal bleeding score			0.15			0.12
0 (normal)	0	0		0	0	
1 (1–2 great than normal)	4	13		4	10	
2 (3–4 great than normal)	21	22		21	20	
3 (≥ 5 greater than normal)	10	20		10	5	
Stool frequency	4.7 ± 0.8	4.8 ± 1.8	0.43	4.7 ± 0.8	5.6 ± 1.1	0.16
CRP(mg/L)	33.1 ± 12.8	31.7 ± 15.0	0.07	33.1 ± 12.8	37.3 ± 13.6	0.23
ESR (mm/h)	30.3 ± 8.6	33.2 ± 12.4	0.08	30.3 ± 8.6	34.2 ± 14.4	0.06
Mayo score	6.9 ± 1.6	6.9 ± 2.0	0.50	6.9 ± 1.6	6.9 ± 1.6	0.67

### Treatment outcomes

After 8 week of treatment, 89% (31/35) of patients achieved clinical response in CBMNC-treated group. Clinical remission and endoscopic mucosal healing was observed in 80% (28/35) and 74% (26/35) of patients respectively. There was no improvement in 5 patients (14%). In addition, the Mayo score decreased from 6.9 ± 1.6 to 2.7 ± 2.0 after treatment (*P* < 0.01); Erythrocyte sedimentation rate (ESR) decreased from 30.3 ± 8.6 mm/h to 14.5 ± 3.9 mm/h after treatment (*P* < 0.01); C-reactive protein (CRP) levels decreased from 33.1 ± 12.8 mg/l to 9.9 ± 4.6 mg/l after treatment (*P* < 0.01); and the defecation frequencies decreased from 4.7 ± 0.8 times per day to 2.7 ± 0.7 times per day after treatment (*P* < 0.01) (Table 2).

**Table 2:** Comparison of clinical efficacy between CBMNC Group and AZA Group 8 weeks after treatment

Parameter	CBMNC Group	AZA Group	*P* value
Clinical response	31/35 (89%)	27/35 (77%)	0.21
Clinical remission	28/35 (80%)	20/35 (57%)	0.04
Mucosal healing	26/35 (74%)	18/35 (51%)	0.048
Mayo scores	2.7 ± 2.0	3.2 ± 1.9	0.23
ESR (mm/h)	14.5 ± 3.9	18.0 ± 5.7	< 0.01
CRP (mg/L)	9.9 ± 4.6	10.3 ± 5.3	0.75
Stool frequency	2.7 ± 0.7	2.6 ± 0.8	0.2

Note: The compare among clinical response, clinical remission and mucosal healing using Chi-square test or Fisher’s exact test; The rest of the part using paired student *t* test, *P* < 0.05 was statistically significant Plus-minus values are mean ± SD

In AZA-treated group, 77% (27/35) of patients showed clinical response, 57% (20/35) achieved clinical remission and 51% (18/35) achieved endoscopic mucosal healing, while no improvement was observed in 8 patients (23%). Moreover, the Mayo score decreased from 6.9 ± 1.6 to 3.2 ± 1.9 after treatment (*P* < 0.01); ESR decreased from 34.2 ± 14.4 mm/h to 18.0 ± 5.7 mm/h (*P* < 0.01); CRP levels decreased from 37.3 ± 13.6 mg/l to 10.3 ± 5.3 mg/l after treatment (*P* < 0.01); and the defecation frequencies decreased from 5.6 ± 1.1 times per day to 2.6 ± 0.8 times per day after treatment (*P* < 0.01). The ratios of clinical remission and endoscopic mucosal healing were markedly higher in CBMNC-treated group compared with those in AZA-treated group (*P* < 0.05). In addition, the level of ESR was found to be decreased in CBMNC-treated group compared to that in AZA-treated group (*P* < 0.01). But there was no significant difference in clinical response ratio, Mayo score, CRP levels and defecation frequency between two groups after 8 week of treatment (all *P* > 0.05).

After 16 week treatment, 71% (25/35) patients achieved clinical remission and 69% (24/35) patients achieved endoscopic mucosal healing in CBMNC-treated group. The level of ESR decreased from 30.3 ± 8.6 mm/h to 16.2 ± 5.0 mm/h. In AZA-treated group, 49% (17/35) achieved clinical remission and 46% (16/35) patients achieved endoscopic mucosal healing. ESR level decreased from 34.2 ± 14.4 mm/h to 18.8 ± 5.3 mm/h. At week 16, CBMNC group still showed high ratios of clinical remission and endoscopic mucosal healing and lower ESR level compared to AZA group (all *P* < 0.05).

After 36 week treatment, 78% (25/32) of patients achieved clinical remission and 69% (22/32) of patients achieved endoscopic mucosal healing in CBMNC-treated group. ESR level decreased from 30.3 ± 8.6 mm/h to 16.9 ± 6.0 mm/h. In AZA-treated group, 52% (16/31) patients achieved clinical remission and 42% (13/31) patients achieved endoscopic mucosal healing. ESR level decreased from 34.2 ± 14.4 mm/h to 19.6 ± 6.1 mm/h. At week 36, CBMNC group still showed high ratios of clinical remission and endoscopic mucosal healing compared to AZA group (*P* < 0.05). The ESR level was lower than that of AZA-treated group, but the difference was not significant (*P* = 0.08).

**Figure 1:**
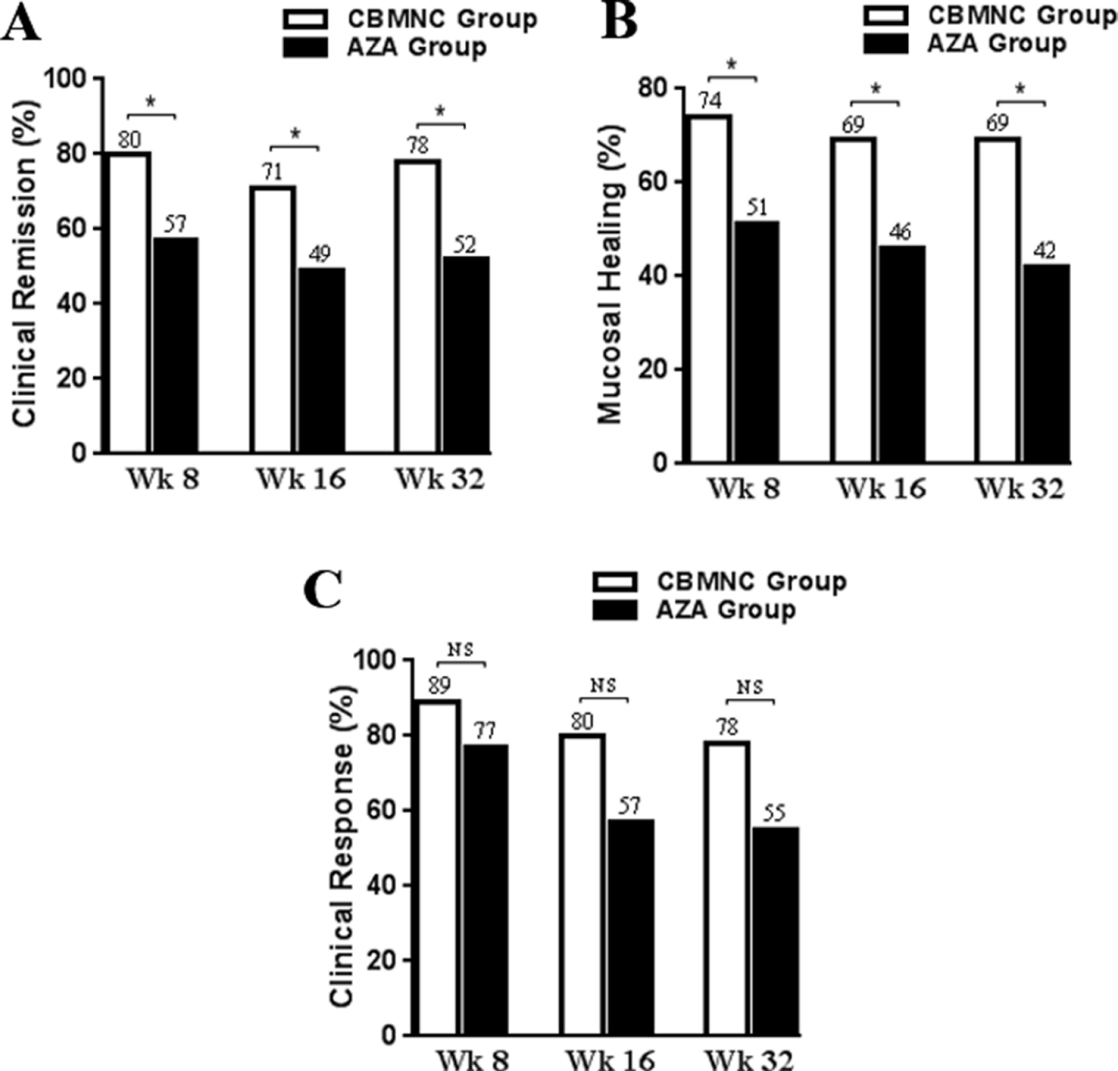
The proportion of patients with a Clinical remission (**A**), Mucosal healing (**B**), and Clinical response (**C**) in CBMNC-treated and AZA-treated Group at weeks 8, 16, and 36. ^*^P < 0.05, NS, no statistical difference, *P* > 0.05.

Although both groups showed improve of clinical remission and endoscopic mucosal healing, the ratios of clinical remission and endoscopic mucosal healing were significantly higher in CBMNC-treated group compared to AZA-treated group at weeks 8, 16 and 36 (all *P* < 0.05, Figure 1).

During and after CBMNC treatment, all patients tolerated well and no major complications occurred. In AZA group, 2 patients (5.7%) suffered from neutropenia and asked to discontinue AZA therapy after 7 month treatment and received leicosen (Jibeier Pharmaceutical Industries, China, registration certificate number: H32025444) (20 mg, tid) therapy; Signs of hepatotoxicity (alanine aminotransferase increase more than 2 times of ULN) were reported in 3 patients (8.6%) after 6 month of treatment, after Polyene Phosphatidylcholine (Sanofi aventis, China, registration certificate number: H20059010) therapy, the level of alanine aminotransferase (ALT) in all 3 patients returned to normal and 2 patients asked to discontinue AZA therapy.

## DISCUSSION

Our results here described that CBMNC therapy appears to be a safe and effective approach for patients with steroid-dependent or -resistant UC. As compared to patients who received AZA therapy, patients who received CBMNC therapy were significantly more like to achieve endoscopic mucosal healing and clinical remission at week 8 and these improvements sustained for 36 weeks after administration. These findings indicate CBMNC as a promising therapy for refractory UC.

The human intestinal epithelium is renewed every 3–5 days by SC residing in the base of crypt which generate about 300 cells per crypt per day. The mucosal lesions in UC patients caused by infiltration of leukocytes and lymphocytes also accompanied by destruction of the crypt structure. Therefore, how to replenish enough SC maybe the key for treatment of UC [5]. In addition, SC especially MSC have the capacity to modulate the immune response through the suppression of dendritic cell maturation and their antigen-presenting abilities [25]. It also demonstrated that human adult SC derived from adipose tissue have the ability to suppress acute inflammatory autoimmune responses by increasing interleulkin-10 and inhibiting inflammatory and Th1 responses in mice [26]. To this end, SC therapies are receiving more attention from IBD investigators.

Hematopoietic SC and MSC have been evidenced to be used for various systemic organ regenerative therapies for almost two decades. With regard to hematopoietic stem cell transplant (HSCT) therapy for IBD, it was reported that 27 IBD cases (22 CD and 5 UC) who underwent HSCT for hematemesis associated cancer from 1993 to 2004 (19 allogeneic and 8 autologous) [27–32]. Among those patients, 24 of 27 patients have achieved clinical remission (0.5 to 8 years) while 3 patients who suffered from relapses underwent autologous HSCT. Burt et al. reported that in the phase I/II study of autologous HSCT (non-myeloablative) in 24 patients with severe refractory CD to anti-TNF therapy, the percentage of clinical relapse-free survival was 91%, 63%, 57%, 39%, and 19% at 1, 2, 3, 4, and 5 years, respectively [33, 34]. These results indicate that CBMNC may act as a promising therapy strategy for the treatment of UC. However, cancer-specific treatment is used for myeloablative treatment in most of above studies which need specified destroy the bone marrow stem cells, resulting in lethal and irreversible marrow failure if SC are not properly reinfused. Non-myeloablative autologous HSCT in autoimmune disease appear to offer substantial benefit compared to myeloablative therapy, but it is still unclear whether autologous HSCT which expand in IBD patients with autoimmune or inflammatory diseases could be fully functional [35]. Moreover, an important finding in a phase III study designated as the autologous HSCT for CD patients has demonstrated that mobilization may be the only benefit of this therapy [24].

For non-myeloablative studies in CD, preliminary results of phase II clinical trial using allogeneic bone marrow-derived MSC for treatment refractory luminal CD also showed prospective efficacy and safety. 12 out of 14 patients were improved, including 8 patients with remission and 7 patients of the endoscopic improvement, and no acute reactions were reported [23]. Therefore, these results highlight the safety and efficacy of CBMNC treatment. In our study, we used non-myeloablative CBMNC therapy for the treatment of UC. *Ex vivo* isolated CBMNC were infused with saline intravenously. After 8 weeks of treatment, CRP and ESR levels which are associated with the activity of UC were significantly decreased, indicating that the CBMNC treatment relieved the inflammatory response in active UC patients. The defecation frequency was also significantly decreased in CBMNC group compared with AZA group. Moreover, clinical remission and endoscopic mucosal healing ratio were significantly higher in the CBMNC group compared with the AZA group. The improved mucosal healing can prevent relapse and improve the life quality of patients. Similarly, these improvements were observed at 16 and 36 weeks after treatment.

In regard to adverse reactions, there were no obvious complications in CBMNC group. These benefits may attribute to the non-myeloablative treatment in this study. In AZA group, side-effects were complained by 5 (14.3%) out of 35 patients which included neutropenia (5.7%) and hepatotoxicity (8.6%). The incidence of side-effects in the AZA-treated group were lower compared with the European studies (31%-45%) [36, 37], this result indicating low frequency of AZA dose-dependent side-effects in these studies. The maintenance dose of AZA was lower in our study (1.5 mg/kg/day) than that in the European study (2–2.5 mg/kg/day) without sacrificing efficacy [38]. The small sample size as well as short follow-up period in our study might be also responsible for low frequency of adverse reactions. Follow-up to week 36, there were no other adverse effects in AZA treatment group.

There were also several limitations in our study. First, it could not avoid selection bias as a retrospective study. Although propensity score-matched analysis was performed to adjust for potential confounding factors, it still did not eliminate unmeasured variables and initial selection bias. A second consideration is our small sample size might not be reflective of a large population and the follow-up period was too short. Besides, the exact mechanisms and long-term adverse effects of this therapeutic remain unknown. Several factors, such as the source and type of SC, the quality control of prepared SC, the procedure of administration (route, dose, pretreatment) and other factors that may influence the transplantation efficacy and how to control the appropriate differentiation in the preferred location are still poorly understood. Despite all of above limitations, this study has shown the potential advantages of CBMNC for the treatment of refractory UC.

In conclusion, CBMNC administration provides effective protection and safety in patients with steroid-dependent or -resistant ulcerative colitis. These patients have higher ratio of clinical remission and mucosal healing at 8 weeks and sustained up to 36 weeks after therapy. Further prospective studies are needed to define the roles of the CBMNC therapy in the treatment of refractory UC.

## MATERIALS AND METHODS

This study was conducted as a retrospective analysis and was approved by the institutional review boards of Chinese PLA General Hospital of Jinan Military Command and The Second Affiliated Hospital of Shandong University of Traditional Chinese Medicine. The requirement to obtain informed consent was waived because of the retrospective nature of this study, but a written informed consent was obtained from each subject before CBMNC therapy after full explanation of the purpose and nature of the procedure used.

### Patient enrollment

**Figure 2:**
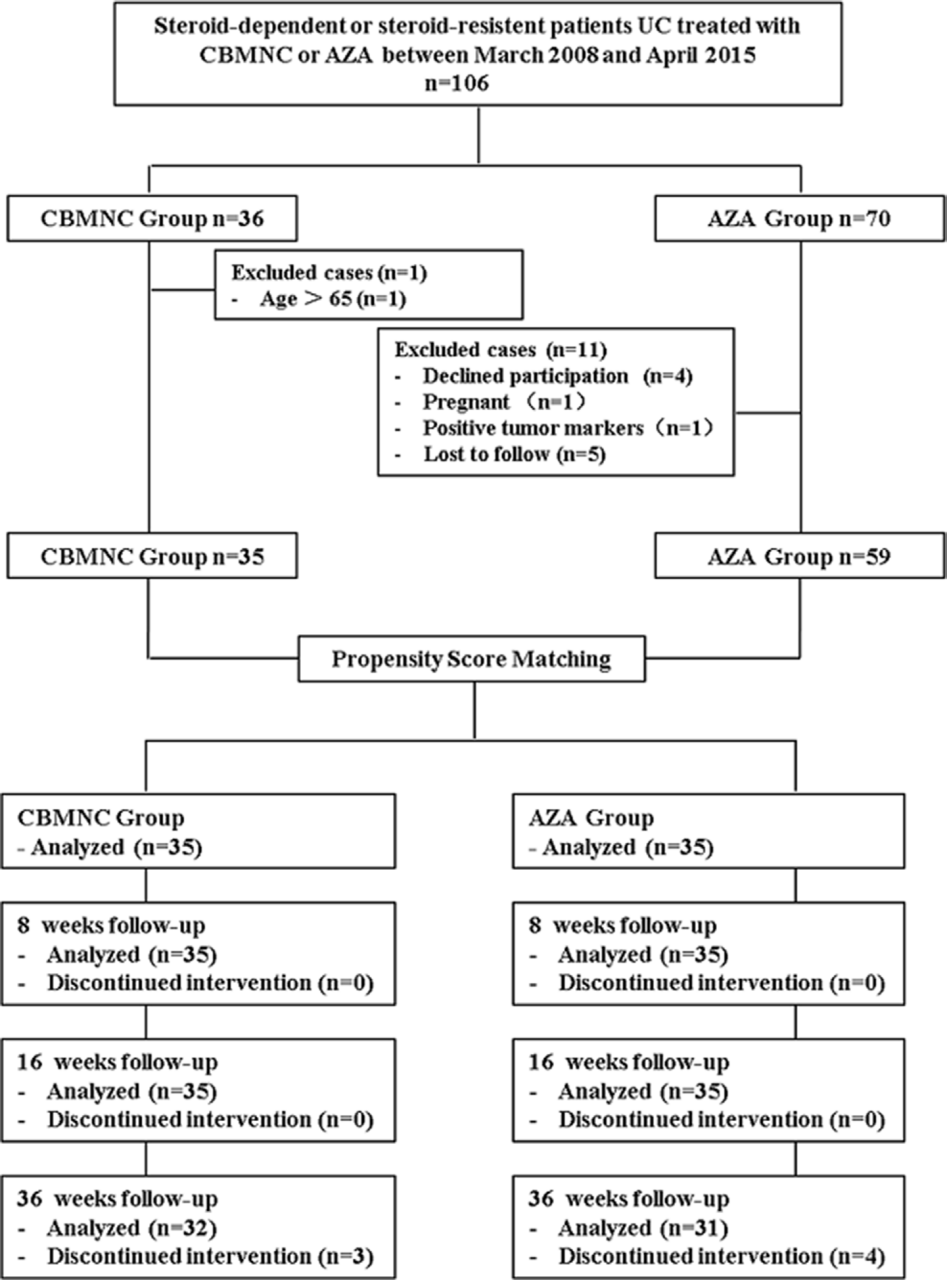
Flow chart summarizes patient inclusion of CBMNC Group and AZA Group CBMNC Group, patients treated with umbilical cord blood mononuclear cells, AZA Group, patients treated with azathioprine.

106 patients from Chinese PLA General Hospital of Jinan Military Command were enrolled in this study between March 2008 and April 2015. Inclusion criteria included: (a) Male or female (18–65 years of age); (b) Diagnosed with steroid-dependent UC or -resistant UC [38]; (c) 3 ≤ Mayo score ≤ 12; (d) Discontinuation of drugs that may influence UC within 2 weeks prior to the study whereas ongoing steroid therapy was permitted. Exclusion criteria included: (a) Severe co-morbidity, such as cardiopulmonary, liver or renal dysfunction, or local and systemic infections; (b) Crohn’s disease; (c) Positive serum HIV or tumor markers; unable or unwilling to sign the consent; (d) Participated in other clinical trials during the last 6 months; (e) Patient underwent a immunosuppressant (AZA, 6-mercaptopurine) treatment within 6 months; (f) Pregnant or lactating women; (g) Patients with incomplete data were also excluded (Figure 2).

### Patient treatment design

Patients with steroid-dependent or -resistant UC patients were assigned into two groups, CBMNC-group and AZA-treated group. Patients in CBMNC group received CBMNC transplantation, while in the AZA group received AZA (ExcellaGmbH, Germany, registration certificate number: H20100042) (1.5 mg/kg, qd). Both groups were given mesalazine (Dr. Falk Pharma GmbH, Germany, registration certificate number: H20100110) (1 g, qid) and maintenance doses of prednisone acetate (Eva Pharmaceutical Industries, China, registration certificate number: H44021207) (steroid-dependent UC, 10 mg/d; steroid-resistant UC, 0.75 mg/kg/d) at the same time. All of the patients were followed and diagnosed through colonoscopy and pathological examination.

### Preparation of umbilical cord blood mononuclear cells and administration

Fresh human umbilical cord blood (80–120 ml) was obtained from 36 informed healthy donors. All of samples were tested for hepatitis B, hepatitis C, and HIV, syphilis and cytomegalovirus, and serum ALT. In briefly, umbilical cord blood was first diluted with normal saline in the ratio of 2:1, then transferred 30 ml of the diluted blood into a new tube containing 15 ml Ficoll and centrifuged for 20 minutes at 750 g. Mononuclear cells were isolated and washed twice with normal saline. After lysis of erythrocytes, the final cell density was adjusted into 1 - 2.5 × 10^8^/ml. The cell viability (≥95%) was determined by trypan blue and sterility test. The proportion of CD34^+^ cells were among 1.5% - 2.3% as determined by flow cytometry.

Each patient received two consecutive CBMNC treatments at 1 week interval. Approximately 1 × 10^8^ CBMNC were mixed with 100 ml normal saline and administered intravenously with electrocardiograph monitoring in 30 minutes.

### Post-treatment follow-up

Clinical remission, clinical response, endoscopic mucosal healing were determined according to the changes of Mayo score and laboratory tests were performed at weeks 0, 8, 16, and 36.

The modified Mayo score was used to evaluate clinical efficacy of these therapies [39]. Clinical response was defined as a decrease of at least 3 points and 30% from baseline (Mayo score), with an accompanying decrease of at least 1 point of subscore for rectal bleeding or absolute subscore for rectal bleeding of 0 or 1. Clinical remission was determined as a total Mayo score ≤ 2 points with no individual subscore > 1 point. An absolute subscore for endoscopy of 0 or 1 was defined for mucosal healing [40].

The laboratory tests include ESR and CRP, whole blood cell analysis, liver and renal function, levels of sodium, chlorine, potassium, serum ALT, creatinine, blood urea nitrogen, and total bilirubin were performed at baseline (pre-treatment) and weeks 8, 16, and 36 after initial treatment. Colonoscopy was performed before treatment and at weeks 8, 16, and 36 during the follow-up in all of patients to observe lesions of the intestinal mucosa including erythema, vascular texture, tissue fragility, erosion, and hemorrhage. Moreover, any adverse events including neutropenia, hepatotoxicity or headaches were recorded during the follow up periods.

### Statistical analysis

The primary end point of this study was clinical remission ratio of these two groups at weeks 8, 16 and 36 after treatment. The secondary end points consisted of clinical response ratio, endoscopic mucosal healing ratio, Mayo score, ESR and CRP levels, and defecation frequency. To decrease confounding bias caused by nonrandomized retrospective assignment, PSM was applied to balance the pretreatment data of two groups. In brief, PSM for all of the patients were evaluated by multiple logistic-regression using variables of sex, age, disease course, disease extent, defecation frequency, ESR, CRP and Mayo score. By using the Nearest-neighbor method, a 1:1 matching study group was created. In the PSM population, qualitative variables were analyzed using Fisher’s exact test or Chi-square test. Normal distribution quantitative data were analyzed by means of one-way analysis of variance. Skew distributional data were compared using Mann–Whitney *U* test. SPSS 22.0 (SPSS, Inc., Chicago, IL, USA) and SPSS Sample Power (version3.0; IBM, USA) were used for statistical analyses. The significance level was defined as *P* value < 0.05.

## Abbreviations

CBMNC: Umbilical cord blood mononuclear cells
AZA: Azathioprine
UC: Ulcerative colitis
CD: Crohn’s disease
IBD: Inflammatory bowel disease
SC: Stem cell
MSC: Mesenchymal stem cells
PSM: Propensity score matching
ALT: Alanine aminotransferase
ESR: Erythrocyte sedimentation rate
CRP: C-reactive protein
(HSCT): Hematopoietic stem cell transplant
